# Enhancing enzymatic saccharification yields of cellulose at high solid loadings by combining different LPMO activities

**DOI:** 10.1186/s13068-024-02485-6

**Published:** 2024-03-09

**Authors:** Camilla F. Angeltveit, Anikó Várnai, Vincent G. H. Eijsink, Svein J. Horn

**Affiliations:** Faculty of Chemistry, Biotechnology, and Food Science, Norwegian University of Life Sciences (NMBU), Ås, Norway

**Keywords:** Lytic polysaccharide monooxygenase, LPMO, AA9, Cellulolytic enzyme cocktails, Enzymatic saccharification, Inactivation, Hydrogen peroxide, High-solids effect

## Abstract

**Background:**

The polysaccharides in lignocellulosic biomass hold potential for production of biofuels and biochemicals. However, achieving efficient conversion of this resource into fermentable sugars faces challenges, especially when operating at industrially relevant high solid loadings. While it is clear that combining classical hydrolytic enzymes and lytic polysaccharide monooxygenases (LPMOs) is necessary to achieve high saccharification yields, exactly how these enzymes synergize at high solid loadings remains unclear.

**Results:**

An LPMO-poor cellulase cocktail, Celluclast 1.5 L, was spiked with one or both of two fungal LPMOs from *Thermothielavioides terrestris* and *Thermoascus aurantiacus, Tt*AA9E and *Ta*AA9A, respectively, to assess their impact on cellulose saccharification efficiency at high dry matter loading, using Avicel and steam-exploded wheat straw as substrates. The results demonstrate that LPMOs can mitigate the reduction in saccharification efficiency associated with high dry matter contents. The positive effect of LPMO inclusion depends on the type of feedstock and the type of LPMO and increases with the increasing dry matter content and reaction time. Furthermore, our results show that chelating free copper, which may leak out of the active site of inactivated LPMOs during saccharification, with EDTA prevents side reactions with in situ generated H_2_O_2_ and the reductant (ascorbic acid).

**Conclusions:**

This study shows that sustaining LPMO activity is vital for efficient cellulose solubilization at high substrate loadings. LPMO cleavage of cellulose at high dry matter loadings results in new chain ends and thus increased water accessibility leading to decrystallization of the substrate, all factors making the substrate more accessible to cellulase action. Additionally, this work highlights the importance of preventing LPMO inactivation and its potential detrimental impact on all enzymes in the reaction.

## Background

There is a critical need for technology that allows efficient utilization of renewable resources like lignocellulosic biomass to combat the environmental effects of human fossil fuel consumption. Lignocellulosic plant biomass is a ubiquitous source of the carbohydrate polymers cellulose and hemicellulose, which may be depolymerized to yield fermentable sugars that can be converted to biofuels and value-added chemicals [1]. Efficient depolymerization of these polysaccharides is hampered by the recalcitrant structure of plant cell walls. At the same time, efficient production of concentrated sugar syrups is essential for cost-effective conversion of lignocellulosic biomass into valuable products [2–5]. Performing enzymatic saccharification processes efficiently at elevated solid loadings is pivotal in reducing the overall expenses associated with lignocellulosic biorefineries, thereby enhancing the feasibility of lignocellulose valorization.

Performing enzymatic saccharification at high dry matter (DM) levels is known to hamper yields and conversion rates, an effect that is referred to as "the high-solids effect" [6]. A high DM content refers to a situation in which little-to-no free water is present at the beginning of a reaction, meaning that the substrate holds all the water [4, 7]. The amount of free water will depend on the substrate composition and pretreatment methods. However, a DM content of 15–20% (w/w) is typically considered "high" [2]. Several studies employing commercial enzyme cocktails predominantly composed of endo- and exo-acting cellulases have demonstrated a linear reduction in the enzymatic conversion yield with increasing substrate concentration [6, 8–14].

A direct consequence of elevated solid loadings is increased slurry viscosity, which hampers adequate mixing. Additional challenges arise from non-productive cellulase adsorption to phenolic compounds [15, 16], enzyme inhibition by compounds like furan derivatives formed during commonly used pretreatment methods such as steam explosion [16], and feedback inhibition of cellobiohydrolases or β-glucosidases (BGs) due to the accumulation of cellobiose or glucose, respectively [17, 18]. Nevertheless, recent literature suggests that water constraints are the most prominent contributor to the high-solids effect [2, 5, 19]. Water has multiple roles during enzymatic saccharification: it functions as a solvent facilitating the contact between enzymes and their substrate, it acts as a reactant during hydrolysis, and it is responsible for the diffusion of products from the site of enzymatic reaction [20]. Despite efforts in the last decades, the challenges posed by high-solids conditions remain a subject of ongoing studies.

Lytic polysaccharide monooxygenases (LPMOs) were discovered in 2010 [21] and are included in current commercial cellulase cocktails [22]. LPMOs are copper-dependent redox enzymes that require a priming reduction and an oxygen species as co-substrate [21], most probably H_2_O_2_ [23], to perform catalysis. The reduced LPMO-Cu(I) complex will oxidatively break the scissile glycosidic bond in cellulose, leading to the formation of an aldonic acid or gemdiol-aldose for C1- or C4-oxidizing LPMOs, respectively [24, 25]. LPMOs are prone to non-reversible inactivation in the presence of excess H_2_O_2_ [23], which can lead to release of the active-site copper that may fuel transition metal-dependent futile side reactions, such as abiotic oxidation of reducing compounds [26, 27]. Numerous studies have shown that LPMOs improve the efficiency of classical hydrolytic cellulases, likely due to LPMOs' ability to attack the more crystalline parts of the polysaccharide substrate [28–34].

Several studies have tried to shed light on the mechanism behind the synergistic relationship between LPMOs and cellulases [34–39], one important outcome being that the oxidative regioselectivity of the LPMOs plays a role. For example, C1-oxidizing LPMOs tend to synergize well with processive cellulases attacking the nonreducing-end, while C4-oxidizing LPMOs seem to have a better effect when combined with cellulases attacking the reducing ends of the cellulose chains [35, 38]. A recent study has shown that the LPMO effect may not be as "direct" as initially suggested. Studies of the effects of LPMO pretreatments showed that the chain ends introduced by LPMO action do not necessarily serve as immediate access points for cellulases. Instead, it was suggested that LPMO promotes time-dependent decrystallization of the substrate that improves accessibility for the classical hydrolytic enzymes [40]. Indeed, several studies support the notion that LPMO action promotes decrystallization of cellulose [41–44]. Recently, Cannella et al. [45] showed that oxidation of filter paper with an LPMO, or chemically, using TEMPO [(2,2,6,6-tetramethylpiperidin-1-yl)oxyl] increases the amount of water retained by the fibers, due to the increased negative surface charge. Thus, LPMO activity will increase the hydrophilicity and water content of the substrate, which could help mitigate the negative effects of high DM conditions on cellulase performance.

While the impact of LPMOs on the efficiency of cellulolytic enzyme cocktails has been studied extensively, little is known about the effect of the DM level and the role LPMOs may play in counteracting the high-solids effect. It is important to note that water availability depends on the DM content and, therefore, that saccharification performances cannot be directly compared across low and high DM experiments [19]. The effect of DM loading (1–15%) on AA9 LPMO activity was recently shown to vary a lot depending on the type of LPMO. Some LPMOs gave more product release as DM content was increased, while other LPMOs seemed to be substrate saturated and even inhibited at high DM [44]. To gain more insight into these matters, in this study, a commercial LPMO-poor enzyme cocktail, Celluclast 1.5L, was spiked with two different fungal AA9 LPMOs, C1-oxidizing *Tt*AA9E from *Thermothielavioides* (previously *Thielavia*) *terrestris* and predominantly C4-oxidizing *Ta*AA9A from *Thermoascus aurantiacus* to investigate the impact of LPMOs on cellulose saccharification at elevated DM concentrations. Using various experimental setups, we show that LPMOs are increasingly important for saccharification efficiency at higher substrate concentrations, notably in a manner that varies between LPMOs and substrates. We also show the importance of preventing LPMO inactivation, not only because LPMO activity is needed, but also because copper leaking out of inactivated LPMOs [27, 46] facilitates unfavorable side reactions.

## Methods

### Steam-exploded wheat straw

Steam-exploded wheat straw was provided by Novozymes. The compositional analysis was performed based on the standard operating procedure developed by NREL [47] and is shown in Table 1. The DM content was measured using Karl Fischer titration as described elsewhere [48] and found to be 52% (w/w). The substrate was stored at − 20 °C.

**Table 1:** Composition of steam-exploded wheat straw

Ash	Arabinan	Galactan	Glucan	Xylan	Mannan	Total lignin
7.70	1.62	0.71	47.48	19.19	0.33	22.51

### Enzymes

*Ta*AA9A from *Thermoascus aurantiacus* and *Tt*AA9E from *Thermothielavioides (*previously *Thielavia) terrestris*, as well as Celluclast 1.5 L, NZ-BG (a β-glucosidase preparation), and Cellic CTec2 were kindly supplied by Novozymes (Novozymes, Bagsværd, Denmark). The protein concentrations were determined using the Bradford method with BSA (Sigma-Aldrich, St. Louis, MO, USA) as standard. Both LPMOs were copper saturated as described previously [49], followed by desalting using a PD MidiTrap column (G-25; GE Healthcare, Chicago, IL, USA). All enzymes were stored at 4 °C.

### Standard reaction setup

The enzyme dosage was held constant at 4 mg protein per g substrate for all reactions. Reactions without LPMO were performed with Celluclast 1.5 L and NZ-BG in a 9:1 ratio (protein:protein). For the reactions supplemented with LPMO, the LPMO constituted 10% of the total protein dose (i.e., 0.4 mg/g substrate). The BG dose was held at 10% of total protein (0.4 mg/g substrate) in all reactions to ensure the complete conversion of cellobiose to glucose. Thus, Celluclast 1.5 L represented 80% of the protein (3.2 mg/g substrate) in reactions with added LPMO and 90% (3.6 mg/g substrate) in reactions without added LPMO. Reactions with Cellic CTec2 were performed without addition of BG at 4 mg protein per g substrate.

The substrates were microcrystalline cellulose (Avicel PH-101, 50 µm particles; Sigma-Aldrich) or steam-exploded wheat straw and reactions were run at 5–25% (w/w) DM concentrations in 50 mM sodium acetate buffer (Sigma-Aldrich), pH 5.0. If not specified otherwise, 10 mM ascorbic acid (Sigma-Aldrich) was added at the beginning of all reactions with Avicel. Glucose feedback inhibition of enzyme cocktails was probed by adding 2.5, 5.0, or 10% (w/w) glucose (Sigma-Aldrich) at the start of the reaction in addition to the cellulose substrate.

### Reaction termination and dilution

All time points (5, 24, 48, and 72 h) were run as individual reactions in 2 mL Eppendorf tubes with 0.6 mL reaction volume in an Eppendorf Thermomixer (Eppendorf, Hamburg, Germany) at 50 °C and 1000 rpm. The reactions were terminated by boiling the samples at 100 °C for 20 min before samples were diluted to 1% DM (w/w) by transferring the whole reaction slurry to 15 mL Falcon tubes and diluting with sodium acetate buffer [6] to minimize errors associated with the higher DM contents [3]. Afterward, the samples were thoroughly mixed, 250 µL of each were filtered with a 96-well filter plate (0.2 µm; Sigma-Aldrich), and filtrates were stored at 4 °C before further analysis.

### Cellulase inactivation by abiotic reactions

A mixture of Celluclast 1.5 L and NZ-BG (9:1 ratio, 0.6 mg protein in total) was preincubated in 50 mM sodium acetate buffer pH 5.0 at 50 °C and 1000 rpm for 24 h in an Eppendorf Thermomixer together with externally added 10 mM H_2_O_2_ (Sigma-Aldrich), 10 mM ascorbic acid, or 0.63 mM Cu(II)SO_4_. The effects of different combinations of H_2_O_2_ or ascorbic acid with Cu(II) and EDTA (6.3 mM; Sigma-Aldrich) were also tested. After the preincubation, the saccharification reaction was initiated by transferring the preincubated cellulase cocktail (450 µL) to Eppendorf tubes containing 150 mg Avicel, yielding a reaction mixture with 25% DM (w/w) and 4 mg protein per gram of substrate. The saccharification reactions were run at the same conditions as for the preincubation reactions for 24 and 48 h after which they were terminated as described above.

### Analysis of soluble native and oxidized sugars

Glucose levels were analyzed by high-performance liquid chromatography (HPLC) using a Dionex Ultimate 3000 (Dionex, Sunnyvale, CA, USA) connected to a refractive index detector 101 (Shodex, Japan) as described previously [29]. The analytical column was a Rezex ROA-organic acid H + (8%) 300 × 7.8 mm (Phenomenex, Torrance, CA, USA), the eluent was 5 mM H_2_SO_4_, the operating temperature was 65 °C, and the flow rate was 0.6 mL/min. Soluble oxidized sugars (Glc1A, Glc4gemGlc and Glc4gemGlc_2_) were quantified by high-performance anion exchange chromatography with pulsed amperometric detection (HPAEC-PAD) using a Dionex ICS-5000 (Dionex) equipped with a CarboPac PA200 column, as previously described [50, 51]. An eluent gradient from 0 to 100% B (A: 100 mM NaOH; B: 1 M NaOAc + 100 mM NaOH), an operational flow of 500 µL/min, and a sample loop volume of 5 µL were used, as described previously [51]. The results were analyzed using the Chromeleon 7 software program (Dionex).

Standards of glucose, cellobiose, and gluconic acid (C1-oxidized, DP1) were purchased from Sigma-Aldrich and diluted as appropriate. Cellobionic and cellotrionic acid (C1-oxidized, DP2-3) [52] and C4-oxidized standards of DP2-3 [29, 51] were produced as described previously using *Mt*CDH from *Myriococcum thermophilum* [52] or *Nc*AA9C from *Neurospora crassa* [53] respectively.

### Statistical analysis

The statistical analysis was performed with a two-tailed Student's t-test using Microsoft Excel (Office 365).

## Results and discussion

### The role of LPMOs at different cellulose concentrations

**Fig. 1:**
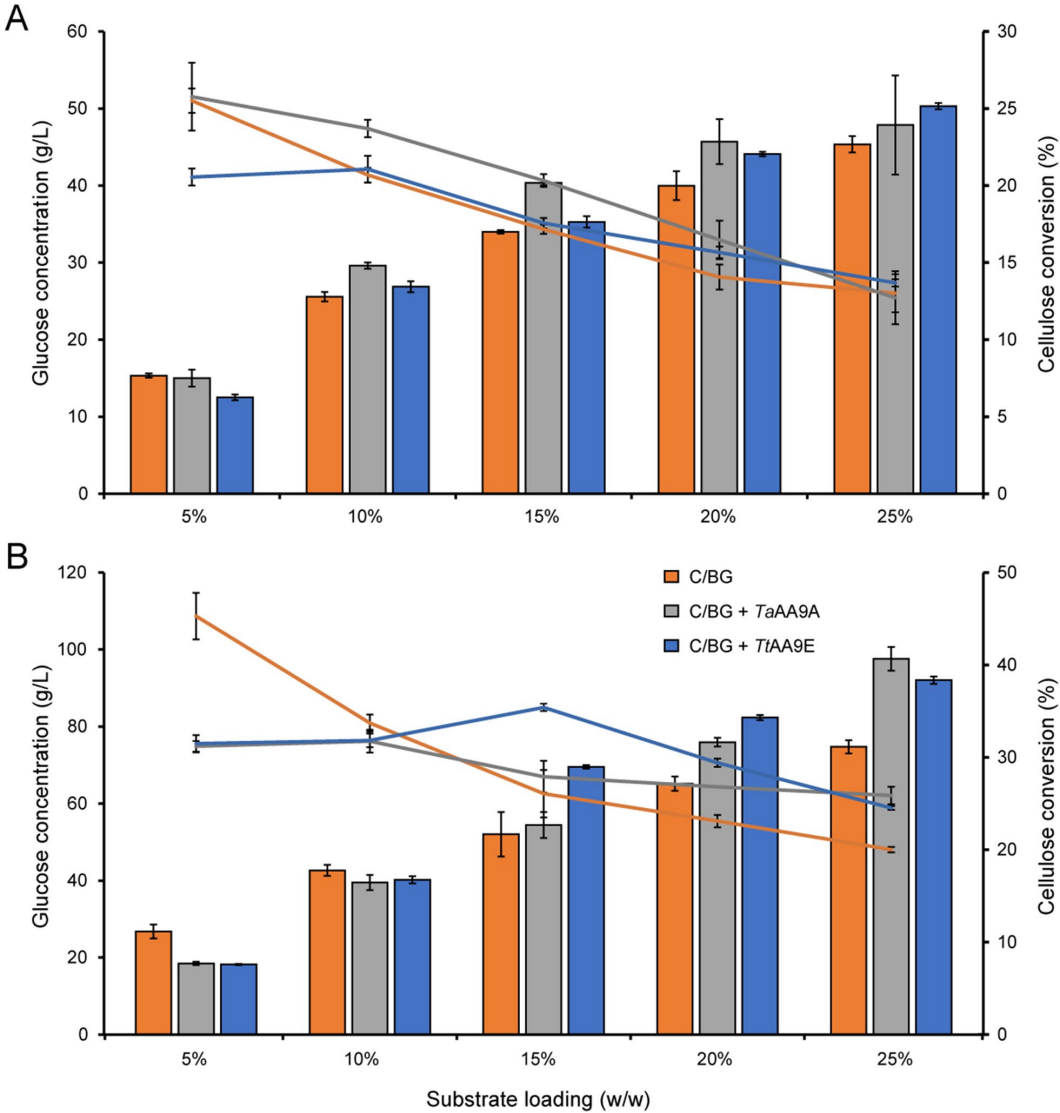
The impact of LPMO supplementation on cellulose saccharification at increasing solids loading. Saccharification reactions containing 5–25% (w/w) Avicel were set up with 3.6 mg/g of Celluclast 1.5 L + 0.4 mg/g NZ-BG or with 3.2 mg/g Celluclast 1.5 L  + 0.4 mg/g NZ-BG + 0.4 mg/g *Ta*AA9A or *Tt*AA9E. All reactions contained 10 mM AscA as reductant. Bars represent the glucose release in g/L (left y-axis), and lines show the percentage of cellulose conversion (right y-axis) after 5 (**A**) and 24 h (**B**). Standard deviations for three biological replicates are shown as error bars

Enzymatic saccharification experiments using Celluclast 1.5 L (supplemented with a β-glucosidase, NZ-BG) with or without LPMOs were run at five different cellulose (Avicel) concentrations ranging from 5 to 25% (w/w). The overall glucose conversion in the reactions with only the cellulolytic enzyme cocktail (90% Celluclast 1.5 L + 10% NZ-BG) decreased with the increasing DM content, and this effect was visible both after 5 and 24 h of saccharification (Fig. 1). Interestingly, for reactions with added LPMOs (80% Celluclast 1.5 L + 10% NZ-BG + 10% LPMO), the high-solids effect was less pronounced after 24 h, as can be seen by comparing the blue and gray line with the orange line in Fig. 1B. This result shows that the importance of the LPMO increases with increasing DM concentrations and saccharification time (Fig. 1), as observed previously [45, 54]. Remarkably, at the lowest substrate concentration (5%), supplementing the cellulolytic enzyme cocktail with LPMOs decreased glucan conversion after 24 h substantially (by about one-third) (Fig. 1B). This result is noteworthy, since it provides an “extreme” illustration of how strongly LPMO effects depend on reaction conditions.

**Fig. 2:**
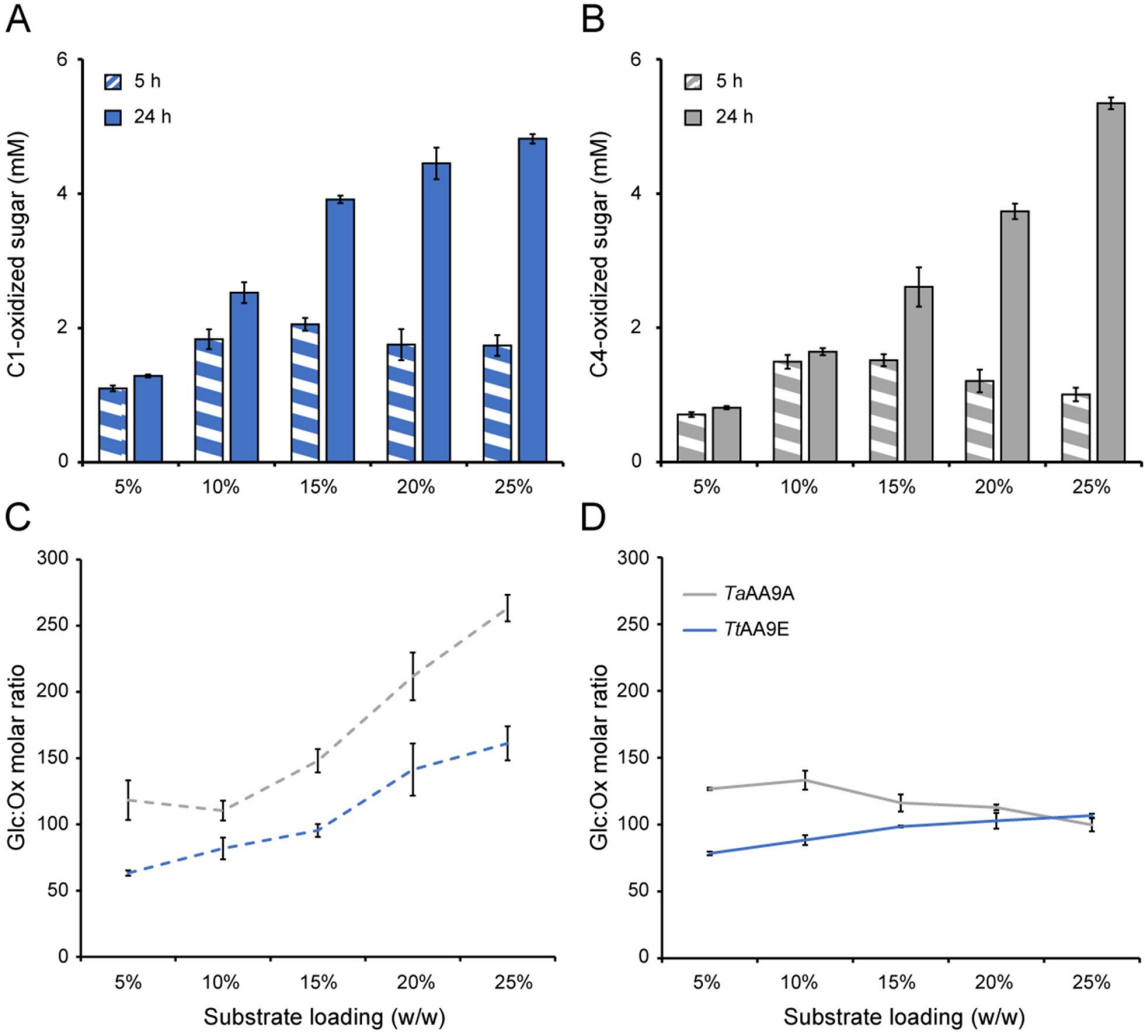
Release of oxidized sugars during saccharification of Avicel at increasing dry matter concentrations. The figure shows the formation of soluble oxidized products in the reactions shown in Fig. 1. Panel **A** shows the soluble C1-oxidized products formed by *Tt*AA9E; panel **B** shows the soluble C4-oxidized products formed by *Ta*AA9A. Panels **C** and **D** show the molar ratio of glucose (from Fig. 1) to total soluble oxidized sugar after 5 and 24 h, respectively. Standard deviations are shown as error bars, for three biological replicates

After 5 h of reaction, the concentration of soluble oxidized LPMO products was highest in the 10 and 15% (w/w) DM reactions. Reactions with higher cellulose concentrations yielded lower concentrations of soluble oxidized sugars (Fig. 2A, B), which could reflect lower LPMO activity or, more likely, that a larger fraction of the oxidized sites remains bound to the substrate (as expected based on the work of Courtade et al. [55]). Similar results have been shown recently for different DM concentrations of cellulose nano-crystals (1–15%), although Magri et al. observed a maximum release of soluble oxidized sugar at 5% DM for the same LPMOs used in our study [44]. However, these experiments were done with LPMOs alone (i.e., no presence of cellulases). Additionally, a recent study has shown that the LPMO oxidation profiles also vary depending on the substrate type [56]. Thus, the results cannot be compared directly.

The ratio of solubilized glucose to solubilized oxidized sugars after 5 h increases with increased DM (Fig. 2C). For the reaction containing *Tt*AA9E, the glucose-to-oxidized sugar ratio increased from 60 to 150 (i.e., approximately 0.7–1.7% of the soluble sugar were oxidized), while for the *Ta*AA9A-containing reaction, the ratio increased from 120 to 260 (i.e., approximately 0.4–0.8% of the soluble sugar were oxidized) when increasing the substrate concentration from 5 to 25% (w/w) (Fig. 2C). After longer incubation, i.e., at 24 h, the concentration of soluble LPMO products (Fig. 2A, B) followed the trends of the glucose concentration (Fig. 1B), meaning that the levels of solubilized oxidized products increased with DM and that the glucose-to-oxidized sugar ratios did not vary much with DM (around 100 for all reactions, i.e., approximately 1% of the soluble sugars were oxidized; Fig. 2D). The fraction of oxidized sugars are similar to that reported in a recent study by Cannella et al. [45], which also observed that the ratio of oxidized to native sugars increased at longer incubation times than 24 h at higher DM levels (10–25%), while the ratio remained stable at the lower DM levels (5%). Although these effects depend on multiple interrelated factors, such as solubilization effects and substrate concentration-dependent effects on LPMO stability, the trends in the levels of soluble oxidized products after 24 h that are visible in Fig. 2 align well with the notion, derived from Fig. 1, that the importance of LPMOs increases at higher DM levels.

In the early stages of saccharification, cellulases work on easily accessible regions of the polysaccharide substrate. As the reaction progresses, the remaining substrate becomes more recalcitrant, exposing regions that are more resistant to enzymatic attack. It is generally believed that LPMOs help break down these recalcitrant structures by introducing oxidative modifications, creating new sites of accessibility that enable cellulases and other enzymes to continue degrading the substrate. Importantly, recent studies indicate that the LPMO–cellulase synergism may be more complex than creating access points [40–43, 45]. The cleavage of a glycosidic bond and concomitant oxidation of the cleavage point allows surrounding water molecules to access the highly ordered fibril structure, leading to decrystallization and amorphization over time [45]. The extent of these larger, and potentially slower, effects will likely vary between C1- and C4-oxidizing LPMOs. Generation of aldonic acids (C1 oxidation) is thought to have the largest effect due to the open ring structure allowing more water to penetrate the crystalline structure [41–43]. On the other hand, recent work by Angeltveit has shown that, with time, the increase in overall accessibility of the substrate for the traditional hydrolytic enzymes will be governed by a time-dependent non-enzymatic decrystallization phase that follows the oxidative action of LPMOs and that does not clearly depend on the oxidative regiospecificity of the enzymes [40]. This aligns with our data showing a significant LPMO effect after 24 h for both the C1- and C4-active LPMOs.

### Increased saccharification efficiency by combining *Tt*AA9E and *Ta*AA9A activity

**Fig. 3:**
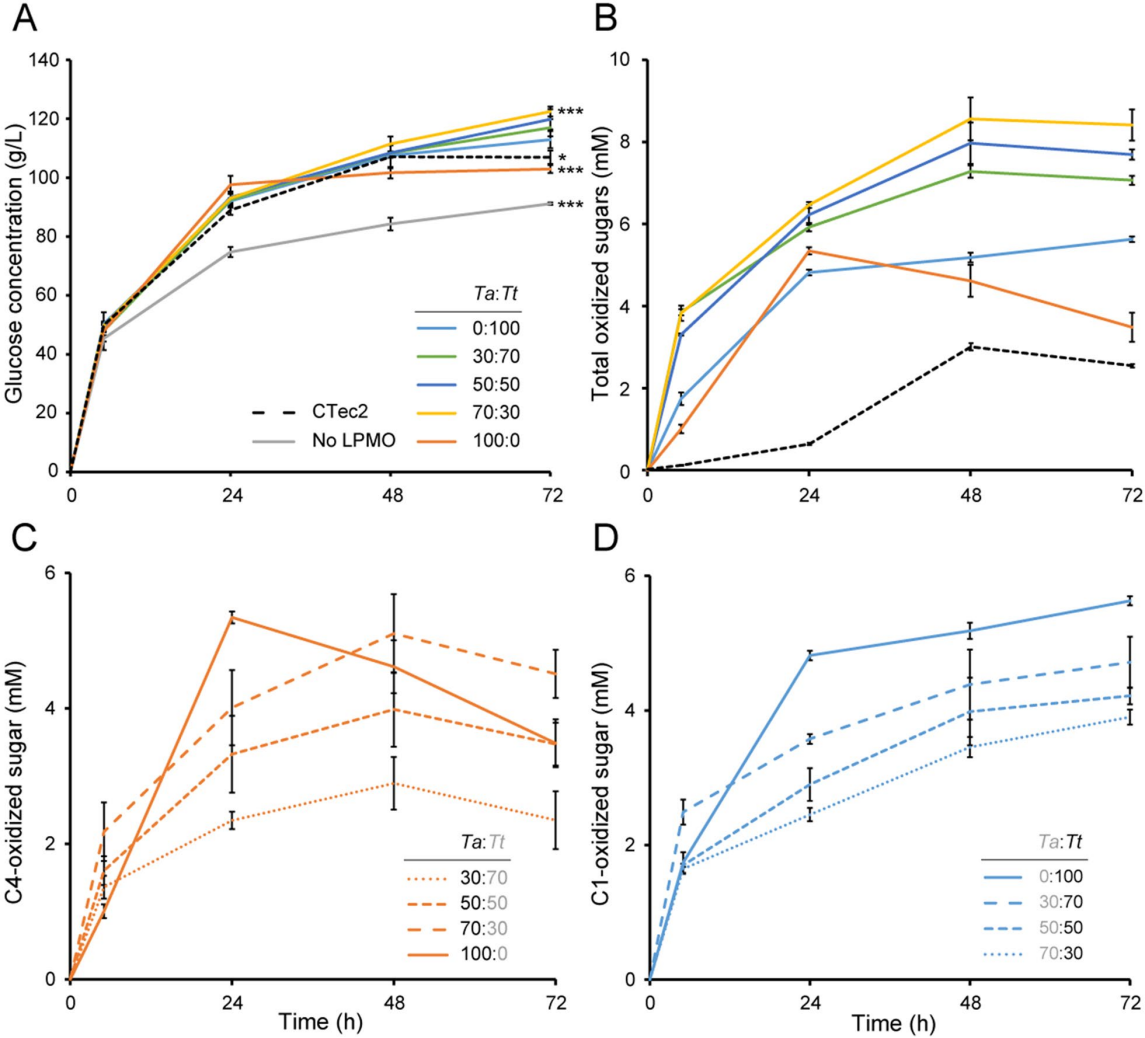
Saccharification of Avicel with LPMO-containing cellulase cocktails at high dry matter. In the reactions, 25% (w/w) Avicel was incubated with either 3.2 mg/g Celluclast 1.5 L + 0.4 mg/g NZ-BG + 0.4 mg/g LPMO (*Ta*AA9A and *Tt*AA9E in varying ratios), or 3.6 mg/g Celluclast 1.5 L + 0.4 mg/g NZ-BG, or 4 mg/g Cellic CTec2. All reactions contained 10 mM AscA as reductant. Panel **A** shows the glucose release; panel **B** shows the total release of oxidized sugars, which is the sum of C4-oxidized products generated by *Ta*AA9A (**C**) and C1-oxidized products generated by *Tt*AA9E (**D**). The symbols * and *** in panel **A** indicate significant differences (p ≤ 0.05 and p ≤ 0.01, respectively) between the cellulase cocktail spiked with *Tt*AA9E only (0:100) and the other enzyme combinations after 72 h (by Student's t-test). Soluble oxidized products were not detected in the reactions without LPMO. Standard deviations are shown as error bars, for three biological replicates

The highest DM content, 25% (w/w), was selected for experiments to investigate the impact of supplementing the reactions with varying ratios of the C1-active (*Tt*AA9E) and the predominantly C4-active (*Ta*AA9A) LPMOs in 72 h reactions with sampling after 5 h and every 24 h. Figure 3A shows a clear positive effect of LPMO inclusion on saccharification yield, with a maximum 38% increase when combining Celluclast 1.5 L with both LPMOs in a 7:3 ratio (*Ta*AA9A:*Tt*AA9E). Early work done prior to the discovery that LPMOs are redox enzymes has shown that each of these LPMOs improves the saccharification of pretreated corn stover, with *Ta*AA9A being the better enzyme [22]. Our results show that, for Avicel, *Tt*AA9E outperforms *Ta*AA9A. It is also worth noting that Celluclast 1.5 L supplemented with any *Tt*AA9E-containing LPMO mixture depolymerized Avicel more efficiently than the more modern LPMO-containing cellulase cocktail Cellic CTec2 (Fig. 3A, B).

The reaction with Celluclast 1.5 L and C4-active *Ta*AA9A showed peculiar kinetics: maximum glucose levels were reached after 24 h (Fig. 3A), and the concentration of C4-oxidized products started declining after 24 h (Fig. 3C). The latter indicates LPMO inactivation and concomitant release of free copper from the active site of oxidatively damaged LPMOs into solution [27]. Under such conditions, i.e., increased availability of H_2_O_2_ due to copper-catalyzed abiotic oxidation of the reductant and accumulation of this H_2_O_2_ because the LPMO no longer consumes it, the C4-oxidized products are unstable and degrade [28]. In reactions with *Tt*AA9E, the levels of C1-oxidized products kept increasing after 24 h (Fig. 3D), indicating that this enzyme stays active longer. In general, LPMO inactivation happens faster at low substrate concentrations [55]. The apparent difference in kinetics and levels of inactivation could be a direct consequence of differences in enzyme stabilities of the two LPMOs or a result of different substrate-binding preferences and thus the experience of different effective substrate concentrations during the reactions. Combining *Tt*AA9E with *Ta*AA9A (and the cellulases) led to an apparent delay in the degradation of C4-oxidized oligosaccharides (Fig. 3C), indicating a moderate stabilizing effect of *Tt*AA9E on *Ta*AA9A for example because *Tt*AA9E still can productively consume available H_2_O_2_. Overall, our data indicate that co-supplementation of *Tt*AA9E and *Ta*AA9A is beneficial, because it leads to less LPMO inactivation and a higher saccharification efficiency.

### The role of LPMOs in enzyme inactivation

In the absence of lignin, like in our reactions with Avicel, LPMOs rely on H_2_O_2_ produced in situ either from abiotic oxidation of the reductant or from the reaction of reduced LPMOs in solution with oxygen [53, 57]. Free (i.e., not substrate-bound) reduced LPMOs lose their activity over time due to oxidative damage to the catalytic site that results from a peroxidase reaction, i.e., futile turnover of H_2_O_2_ [23, 58]. Thus, LPMO stability during a reaction depends on a combination of the level of available H_2_O_2_ and the effective substrate concentration. Of note, when using reductants whose abiotic oxidation is promoted by transition metals such as copper, such as ascorbic acid, LPMO inactivation may be a self-reinforcing process [27]: damage to the catalytic center leads to copper release, which again promotes production of H_2_O_2_, which again promotes LPMO inactivation.

**Fig. 4:**
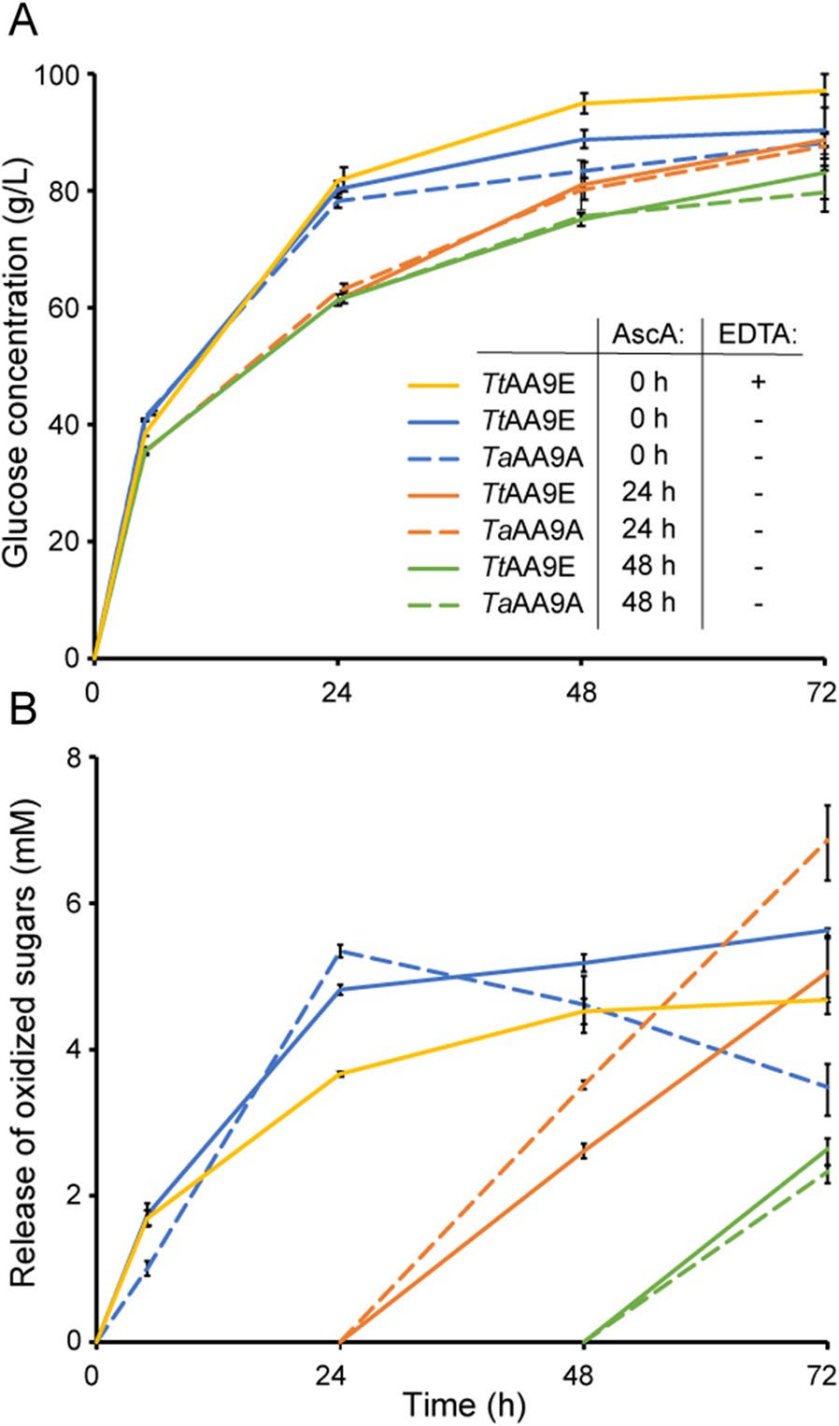
Initiating LPMO activity by adding ascorbic acid at different time points. In the reactions, 25% (w/w) Avicel was incubated with 3.2 mg/g Celluclast 1.5 L + 0.4 mg/g NZ-BG + 0.4 mg/g of either *Ta*AA9A or *Tt*AA9E. The LPMO activity was initiated by adding AscA (10 mM) at different time points. If added, EDTA was present at 6.3 mM. Panel **A** shows glucose release; panel **B** shows the release of soluble oxidized sugars. Standard deviations are shown as error bars, for three biological replicates

Considering the above, we tested whether it could be beneficial to delay reduction of LPMOs and generation of H_2_O_2_ by adding ascorbic acid at specific time points later than 0 h, thus increasing the chance of keeping the LPMOs functional during the later phase of the reaction. The results show that, for the setup used here, delaying the reduction of the LPMOs was not beneficial (Fig. 4). Addition of ascorbic acid at the beginning of the reaction gave, as expected, the fastest initial glucose solubilization. Solubilization yields after 72 h were similar for reactions in which ascorbic acid was added at 0 or 24 h and reduced for the reaction in which ascorbic acid was added after 48 h. These results support the theory of a time-dependent amorphization of the material following the LPMO oxidation rather than the direct creation of access points, and hence, overall making the substrate more accessible for the cellulases.

Non-sufficient removal of unbound copper from the LPMO preparation after copper saturation, "copper-polluted" substrates, and copper leakage from the active site of inactivated LPMOs will influence the activity and inactivation of LPMOs [27, 46, 59]. Copper will speed up production of H_2_O_2_ through abiotic oxidation of ascorbic acid [60] and production of hydroxyl radicals through Fenton-like reactions [61]. To assess possible copper effects, we used ethylenediaminetetraacetic acid (EDTA), which is a good chelator and, hence a scavenger of divalent metal cations such as Cu(II). The dissociation constant for Cu(II) binding by EDTA is between 10^–6^ M and 3.1 · 10^–16^ M [62], i.e., quite similar to published *K*_d_ values for LPMOs, which are in the order of 1 nM for Cu(I) and 50 nM for Cu(II) [63–65]. Addition of 6.3 mM EDTA to a reaction with Celluclast 1.5 L and *Tt*AA9E led to a slight decrease in apparent LPMO activity (Fig. 4B), which may be due to reduced levels of available H_2_O_2_ as a result of reduced levels of transition metals in the reaction solution. Interestingly, despite the lower LPMO activity, the presence of EDTA was beneficial for the overall saccharification yield after 48 h; however, no significant effect was observed after 72 h (Fig. 4A). This suggests that chelation of free copper by EDTA may play a role in preventing additional side reactions that otherwise would damage the enzymes during the course of the reaction.

**Fig. 5:**
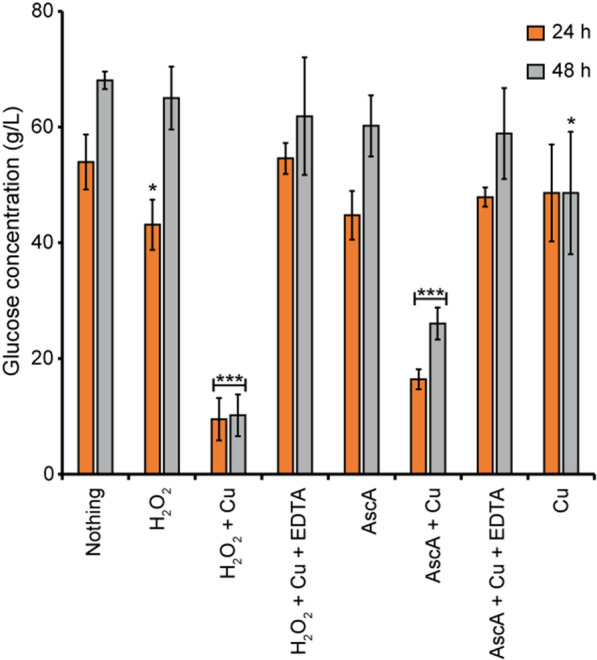
Preincubation of Celluclast 1.5 L prior to Avicel degradation. A 90% Celluclast 1.5 L + 10% NZ-BG mixture was preincubated at 50 °C for 24 h in the presence of H_2_O_2_ (10 mM), AscA (10 mM), Cu(II)SO_4_ (0.63 mM), and/or EDTA (6.3 mM). Following the preincubation, the saccharification reactions were initiated by adding 25% (w/w) Avicel to the preincubated cellulase cocktails, followed by incubation for 24 or 48 h under the same conditions as for the preincubation. The symbols * and *** indicate significant differences (*p* ≤ 0.05 and  *p*≤0.01, respectively) between no preincubation and the different preincubation conditions (by Student's t test). Standard deviations are shown as error bars, for three biological replicates

To gain a deeper insight into the potential impact of abiotic reactions involving ascorbic acid, H_2_O_2_, and free copper on the inactivation of cellulases, Celluclast 1.5 L was preincubated with various combinations of ascorbic acid, H_2_O_2_, Cu(II)SO_4_, and EDTA for 24 h before initiating a saccharification reaction by the addition of Avicel. In general, no significant effects from preincubation with 10 mM H_2_O_2_, 10 mM ascorbic acid, or 0.63 mM Cu(II) alone were observed, except for the 24 h reaction with H_2_O_2_ pretreatment and the 48 h reaction with Cu(II) pretreatment (Fig. 5). However, when H_2_O_2_ or ascorbic acid was combined with Cu(II) during the preincubation, the 24 h conversion yield dropped to only 18% and 30%, respectively, compared to the yields obtained with the cellulase mixture that had not been exposed to any of these compounds. Incubating the cellulase mixture with H_2_O_2_ and free copper had the strongest impact on the cellulase mixture: next to giving the strongest reduction in the 24 h conversion yield, all cellulase activity was lost at this point. Although the applied concentrations of H_2_O_2_ and Cu(II) are higher than what would be seen in the enzyme reactions, a similar molar ratio of these compounds could be expected with H_2_O_2_ concentrations probably being lower than 100 µM [66]. The detrimental effect of H_2_O_2_ and free copper was counteracted by the addition of EDTA, which completely restored the activity of the cellulase cocktail (Fig. 5).

Excess levels of H_2_O_2_ have been shown to inactivate both LPMOs and cellulases [23, 28, 67]. The present results show that the enzymes are relatively stable in the presence of high H_2_O_2_ concentrations (10 mM) as long as transition metals are absent (Fig. 5). Adding copper ions to the system leads to the production of reactive oxygen species such as superoxide and hydroxyl radicals. Thus, observations that seem to indicate that autocatalytic inactivation of LPMOs is accompanied by decreased cellulase activity [28], do not relate only to high H_2_O_2_ levels. Instead, this phenomenon likely arises from side reactions triggered by copper leakage from inactivated LPMOs combined with elevated H_2_O_2_ levels. As a result, the inactivation of LPMOs has significant implications on reaction kinetics and yields.

### Cellulase feedback inhibition

**Fig. 6:**
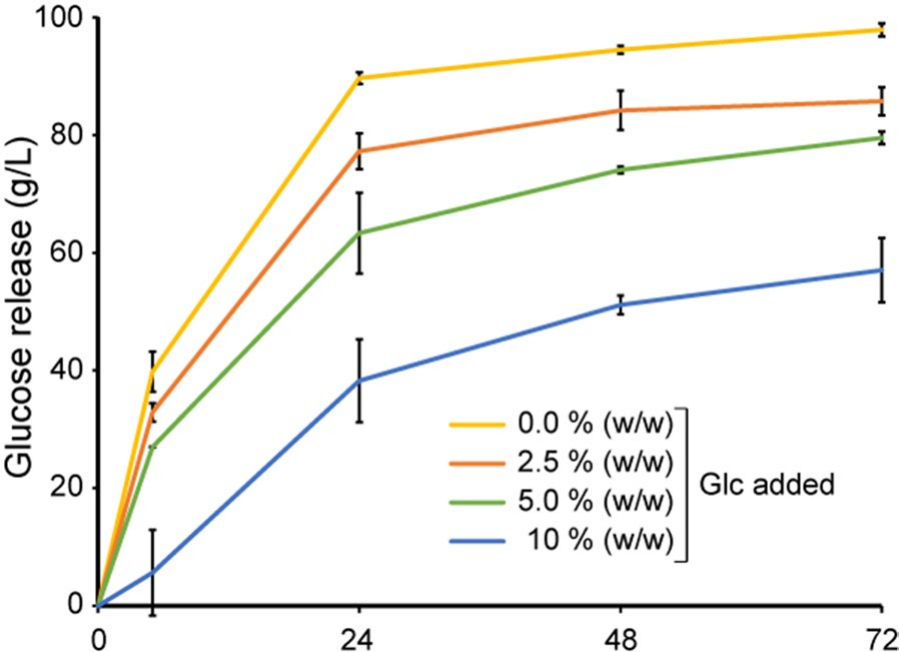
Probing feedback inhibition by glucose. External glucose, up to 10% (w/w), was added to reactions containing 25% (w/w) Avicel and 3.2 mg/g Celluclast 1.5 L + 0.4 mg/g NZ-BG + 0.4 mg/g *Ta*AA9A and *Tt*AA9E in a 1:1 ratio. The figure shows the net glucose release where the externally added glucose concentrations have been subtracted. Standard deviations are shown as error bars, for three biological replicates

It is well established that the initial substrate loading and the accumulation of products during the reaction, i.e., feedback inhibition, influence the saccharification rate, where high concentrations of cellobiose and glucose are known to be inhibitory for cellobiohydrolases and β-glucosidases, respectively [18, 68, 69]. In the present study, Celluclast 1.5 L was supplemented with BG to ensure complete conversion of cellobiose to glucose, and as expected, cellobiose levels in cellulose hydrolysates were negligible. To probe a possible effect of accumulating glucose levels on the saccharification efficiencies described above, cellulose saccharification reactions were carried out with the Celluclast 1.5 L + NZ-BG cocktail spiked with *Ta*AA9A:*Tt*AA9E in a 1:1 ratio in the presence of externally added glucose (Fig. 6). The result shows approximately 10, 20, and 40% decrease in glucose release after 72 h when 2.5, 5.0, and 10% (w/w) glucose was included in the reactions from the start, respectively. The results presented illustrate the high-solids effect and show that glucose feedback inhibition plays a role.

However, several studies suggest that the high-solids effect primarily stems from rate-limiting reorganization of constrained water at the substrate surface upon enzymatic removal of soluble sugars and oligosaccharides [2, 5, 68, 70]. Water coordinating the released soluble mono- and oligosaccharides will take away water from the surface of the insoluble substrate, leading to limited availability of water at the site of catalysis and, consequently, lower enzymatic reactivity. As outlined above, it is conceivable that the substrate polarity and decrystallization that follow LPMO action contribute positively to water accessibility near the site of cellulase catalysis and show that LPMO action is important for overcoming the negative impact of high substrate concentrations. Of note, it has recently been shown that LPMOs are not inhibited by high glucose concentrations [45].

### Saccharification efficiency of steam-exploded wheat straw

The high-solids effect, i.e., a decrease in saccharification efficiency at increasing substrate concentrations, is not only enzyme-dependent (as shown in Fig. 1) but also substrate-dependent. Yields at low- and high-solids concentrations do not correlate for a given biomass, and, thus, industrial evaluation of biomass saccharification should be carried out at high-solids conditions and with the target feedstock [19]. Therefore, we assessed the efficiency of the studied cellulase–LPMO cocktails on a commercial lignocellulosic feedstock, steam-exploded wheat straw provided by Novozymes, at 15% (w/w) substrate loading. Compositional analysis of the steam-exploded wheat straw showed that the feedstock contains around 22% (w/w) hemicelluloses, 22% (w/w) lignin, and 8% (w/w) ash in addition to 48% (w/w) glucan (Table 1).

**Fig. 7:**
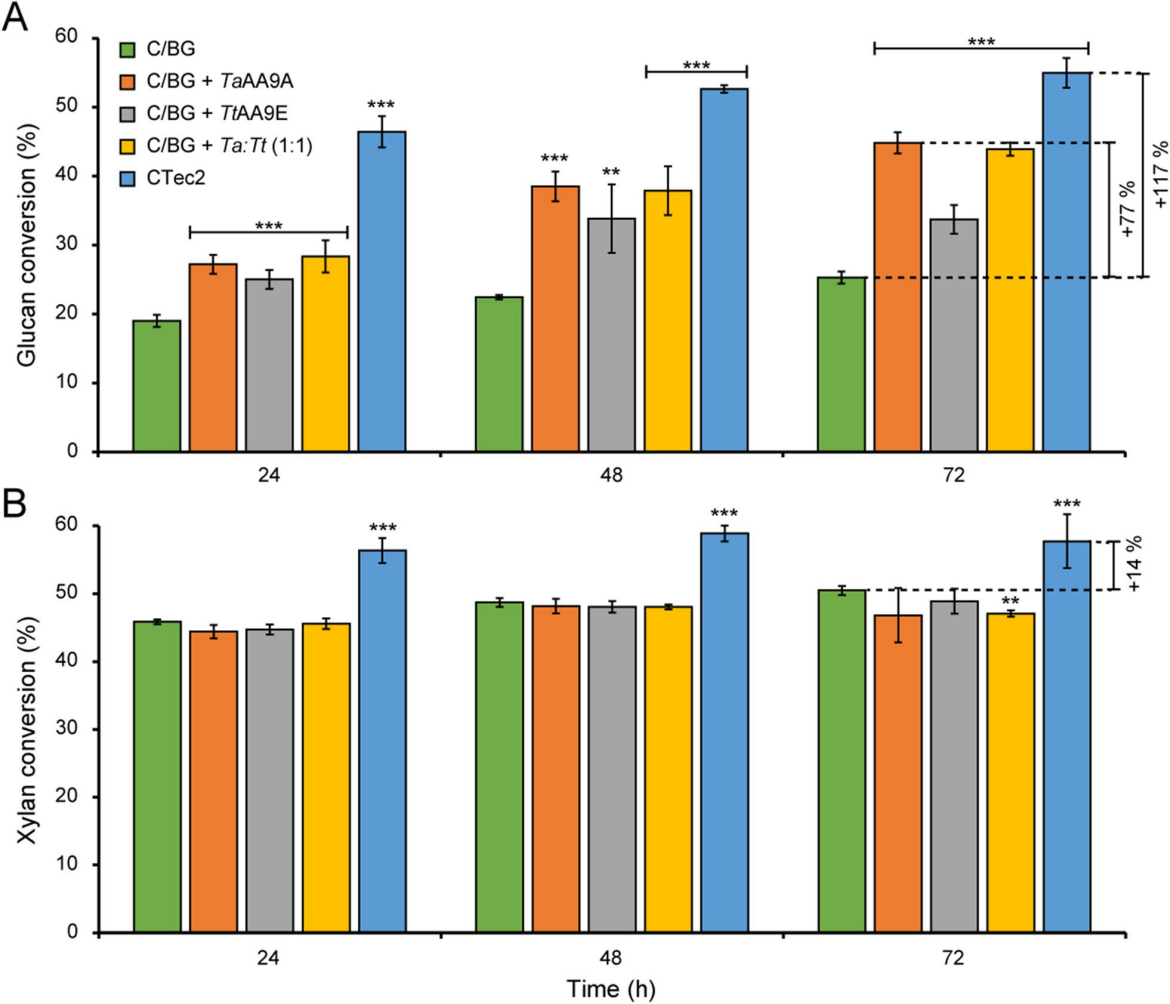
Degradation of steam-exploded wheat straw with various cellulolytic enzyme cocktails. The degradation of 15% (w/w) steam-exploded wheat straw was performed by incubation with either 3.6 mg/g Celluclast 1.5 L + 0.4 mg/g NZ-BG or with 3.2 mg/g Celluclast 1.5 L + 0.4 mg/g NZ-BG + 0.4 mg/g *Ta*AA9A, *Tt*AA9E or a 1:1 *Ta*AA9A:*Tt*AA9E mixture, or with 4 mg/g Cellic CTec2. Panel **A** shows glucan solubilization; panel **B** shows xylan solubilization. The symbols ** and *** indicate significant differences (*p* ≤ 0.025 and *p* ≤ 0.01, respectively) between Celluclast 1.5 L/NZ-BG and Celluclast 1.5 L/ NZ-BG spiked with LPMO(s) or Cellic CTec2 (by Student's t-test). Standard deviations are shown as error bars, for three biological replicates

The results of the saccharification reactions showed that the cellulase cocktail with 10% LPMO inclusion led to drastically increased cellulose solubilization. In this case, *Ta*AA9A, rather than *Tt*AA9E in the case of Avicel (Fig. 3A), had the largest effect: replacing 10% of the Celluclast 1.5 L + NZ-BG cocktail by *Ta*AA9A alone or by a 1:1 mixture of *Ta*AA9A and *Tt*AA9E improved the saccharification by about 75% both after 48 and 72 h (Fig. 7A). On the contrary to the Avicel reaction spiked with* Ta*AA9A, where the glucose release stopped after 24 h, a prolonged period of sugar release was observed in the wheat straw reactions. This shows that the LPMOs are even more important for cellulose solubilization when working with wheat straw at high solid loadings and that the choice of an optimal LPMO is substrate-dependent. The latter conclusion was also reached by Kim et al., in a 2017 study with 1–5% substrate loadings [71].

Xylan solubilization was not affected by replacing 10% of the cellulase cocktail, which includes xylanases, by LPMO (Fig. 7B). Although *Tt*AA9E has been shown to be active on cellulose-bound xylan [72], this activity did not have an apparent effect on the xylan conversion. While the efficiency of the Celluclast 1.5L + NZ-BG + LPMO cocktails surpassed that of Cellic CTec2 in reactions with pure cellulose (Avicel, containing about 1% (w/w) xylan [73]) (Fig. 3A), Cellic CTec2, a modern enzyme cocktail with improved hemicellulolytic activity and with LPMOs included, was more efficient on the xylan-rich wheat straw, releasing higher amounts of glucose and xylose throughout the saccharification reaction (Fig. 7). This aligns well with a study by Hu et al., who showed that supplementation of Celluclast 1.5 L with both xylanases and *Ta*AA9A is required to reach similar levels of cellulose saccharification of steam pretreated pine as when using Cellic CTec2 [32]. Of note, literature speculates that *Ta*AA9A is the dominant LPMO in Cellic CTec2 [29, 32].

## Conclusion

In recent years, multiple studies have addressed the interplay between LPMOs and cellulases. Many of these studies were done with low substrate concentrations, limiting their direct applicability to real-world high-solids processing scenarios. Our study addresses the challenges associated with high-solids systems and shows the pivotal role of LPMOs in cellulolytic enzyme cocktails operating at high DM reactions that run over 24–72 h. Our results show that the positive impact of LPMOs increases throughout the reaction and with increasing DM concentrations.

Accumulating data in studies cited above suggest that the positive LPMO effect is multi-faceted. The increased importance of LPMOs late in saccharification reactions may be attributed to the increasing recalcitrance of the remaining substrate during the reaction, as well as to the relatively slow impact of oxidized cleavage sites on the substrate hydrophilicity and decrystallization. As to negative effects of the presence of LPMOs, recent discoveries highlight the potentially detrimental effects of copper leakage from damaged LPMOs, which may facilitate several side reactions. Our findings demonstrate that maintaining LPMO activity is crucial for the overall saccharification efficiency, not only because LPMO activity is useful, but also because free copper in solution results in detrimental side reactions with H_2_O_2_ that may damage all enzymes in the reaction. Using a different experimental approach and unaware of the fact that LPMOs catalyze productive peroxygenase and potentially damaging peroxidase reactions Scott et al. [67] reached a similar conclusion.

Importantly, our study shows that LPMO effects differ between C1- and C4-oxidizing LPMOs in a DM- and substrate-dependent manner. Thus, despite substantial research efforts in the past decades, there remains a necessity for further optimization and customization of enzyme cocktails tailored to individual feedstocks with specific compositions to attain economically sustainable lignocellulose valorization.

## Data Availability

All data generated or analyzed during this study are included in the published paper.

## Abbreviations

AscA: Ascorbic acid
BG: β-Glucosidase
BSA: Bovine serum albumin
C: Celluclast 1.5 L
DM: Dry matter
DP: Degree of polymerization
EDTA: Ethylenediaminetetraacetic acid
Glc: Glucose
HPAEC-PAD: High-performance anion exchange chromatography with pulsed amperometric detection
HPLC: High-performance liquid chromatography
LPMO: Lytic polysaccharide monooxygenase
NZ-BG: Novozymes β-glucosidase
